# Intensity control of robot-assisted gait training based on biometric data: Preliminary study

**DOI:** 10.1097/MD.0000000000030818

**Published:** 2022-09-23

**Authors:** Kim Jiae, Min Ho Chun, Junekyung Lee, Jun Won Kim, Ji Yeon Lee

**Affiliations:** a Department of Rehabilitation Medicine, Asan Medical Center, University of Ulsan College of Medicine, Republic of Korea; b Department of Rehabilitation Medicine, Hallym University Dongtan Sacred Heart Hospital, Hallym University College of Medicine, Hwaseong, Republic of Korea.

**Keywords:** exercise therapy, gait, robotics, stroke

## Abstract

This study is aimed to compare the effect of robot-assisted gait training when the intensity is controlled using patients’ biometric data to when controlled by therapist’s subjective judgment.

**Design:**

This is non-blinded, prospective, randomized controlled study. Patients were randomly assigned to one of two groups. In biometric data control group, exercise intensity was controlled through the patient’s heart rate or rating of perceived exertion (RPE). The intensity was raised to the next level when the patient’s heart rate reserve was less than 40 percent or the RPE was less than 12 points. The exercise intensity of the therapist control group was adjusted according to the judgement of a therapist. All patients were instructed to perform robot (Morning Walk®)-assisted 20-minute gait training session five times a week during 3 weeks. The primary outcome was functional ambulation category (FAC). The secondary outcomes were modified Barthel index (MBI), Berg balance scale (BBS), timed up and go test (TUG) and 10-meter walk test (10MWT) The outcomes were evaluated at baseline and after 3-week gait training.

**Results:**

A total of 55 patients with stroke were enrolled. After robotic rehabilitation, the primary outcome, FAC improved significantly (*P* < .05) in both groups. Also, secondary outcomes, including MBI, BBS, TUG, 10MWT, showed significant improvement (*P* < .05) in all groups. In addition, when comparing the functional change from baseline to week 3 between the two groups, there was no statistically significant difference in FAC (*P* > .05). The difference of baseline and week 3 of secondary outcome measure, MBI, BBS, TUG, 10MWT, showed no significant difference (*P* > .05).

**Conclusion:**

In conclusion, when the robot intensity was adjusted using the patient’s heart rate or RPE, the treatment effect has no significant difference to when adjusting the intensity according to the know-how of the therapist.

## 1. Introduction

Research into robotic rehabilitation has grown rapidly, and the number of therapeutic rehabilitation robots has increased dramatically during the last two decades. Many reports have described the efficacy of robot-assisted rehabilitation therapy for improving motor and ambulatory function in patients with stroke.^[1,2]^ Robotic devices have been developed to relieve physical therapists from the strenuous burden of manual training.^[3]^

It has been reported that robot-assisted gait training devices can be effective complements to conventional physiotherapy in subacute stroke patients.^[3–5]^ Previous study confirmed that Morning Walk®, gait training robot, improved the gait disturbance in stroke patients.^[6]^

Also intensity of patients’ motor training determine the outcome of the patients motor recovery,^[7,8]^ therefore the need of individualized intensity control appropriate to each patient is being raised. Previous studies recommends prescribing exercise intensity based on rating of perceived exertion (RPE) scale or % heart rate reserve (HRR).^[9]^

Adjusting the intensity according to the know-how of therapists is subjective and depends on the therapist. So, the intensity might not be properly adjusted to patients and usually the quality of the gait training depends on skill of the therapist. Therefore, we can suggest that for effective robotic-assisted gait rehabilitation, the protocol of intensity control based on objective data is needed to be established. However, there have been few attempts to apply a patient’s biometric data to robot-assisted gait rehabilitation. This study is aimed to compare the outcome of robot assisted gait training when the intensity is controlled using patients’ biometric data to when controlled by therapist’s subjective judgment.

## 2. Methods

### 2.1. Study design

This study was an prospective, un-blinded, randomized controlled trial. The trial was conducted at Asan Medical Center from August 2018 to March 2020. It was approved by the Asan Medical Center Institutional Review Board (IRB), No. 2018-1030. This study was registered at the Clinical Research Information Service (KCT0005827). The funding organizations had no involvement in the analysis of the data or the writing up of the paper.

### 2.2. Subjects

We enrolled inpatient stroke patients admitted to the department of Rehabilitation Medicine, over the age of 19 years, who had a gait disturbance or hemiparesis (with a duration of more than 1 week and less than 1yr since stroke). The presence of stroke was confirmed by CT/MRI image. We excluded patients with severe cognitive impairment to the extent that one-step instructions could not be performed. In addition, patients who had difficulty keeping their body and could not carry out robot rehabilitation were excluded. Those who were taking beta-blocker or whose vital sign was unstable and medical treatment required were excluded. In addition, patients were excluded who had severe musculoskeletal disease which affect walking; severe limb contracture or deformity; psychological instability; body weight >135 kg; height >195 cm; an open wound, fracture, or pressure ulcer; the risk of compression fracture due to severe osteoporosis; or contact infection.^[6]^

### 2.3. Procedure

All patients were instructed to perform Morning Walk®-assisted gait training for 20 minutes five times a week for 3 weeks. All participants were provided written informed consents, and they were randomly assigned to one of two groups in a 1:1 ratio. The random assignment was performed by using a random number table. In one group (biometric data control group), exercise intensity was controlled based on the patient’s HRR or RPE, and the other group (therapist control group) were adjusted for exercise intensity according to the know-how of the therapist only.

The Morning Walk®, an end-effector type gait training robot, was used in this study. Hyundai Heavy Industries and Taeha Mechatronics in Korea developed this robot, and the Food and Drug Administration approved the device in December 2014. For patients who cannot stand independently, it provides saddle so that the patients can safely get gait training.

The participants started with ground-level gait and progressed to up and down stair gaits. Various parameters of ground walking such as cadence, step length, step height, initial contact angle, and toe-off angle can be adjusted individualized to each patient. In addition, it provides the graphical information on the monitor as visual biofeedback to the patient for forming good center of pressure pattern.^[6]^

**Figure 1.**
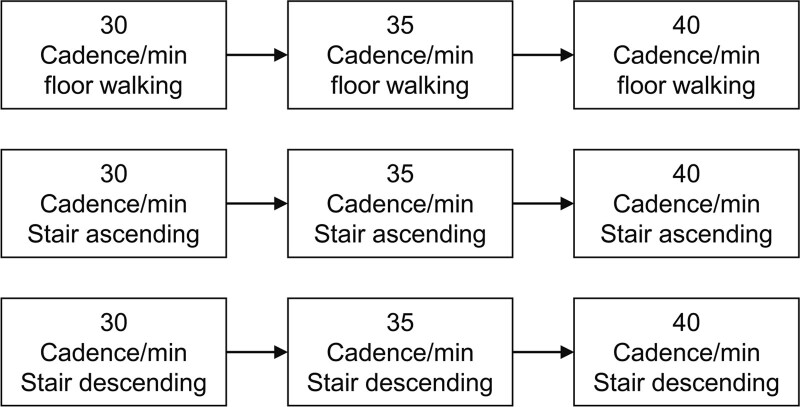
**Morning walk Protocol**. All patients started robotic rehabilitation at a rate of 30 cadence/minute on a flat slope (floor waking). Then, the walking speed was slightly increased to 35, and 40 cadence/minute gradually. The harder intensity is achieved by tilting up the table (stair ascending mode) at 30 cadence/minute. At stair ascending mode, the walking speed can be raised to 35, or to 40 cadence/minute step by step. After that the intensity can be raised by tilting down the table, stair descending mode. The stair descending mode starts from 30 cadence/minute and can be adjusted to 35, and the most strenuous 40 cadence/minute stair descending mode.

Regardless of the patient’s function level, all patients started robotic rehabilitation at a rate of 30 cadence/minute on a flat slope (floor waking). If the patient could tolerate at a rate 30 cadence/minute, the walking speed was slightly increased to 35, and 40 cadence/minute gradually. If the target heart rate was not achieved at a faster speed, the intensity was increased by tilting the table (stair ascending). So, the intensity in Morning Walk® can be raised to 30 cadence/minute, stair ascending mode. At stair ascending mode, the walking speed can be raised to 35, or to 40 cadence/minute step by step to reach the target heart rate. If it is not sufficient to patient, the intensity can be raised by tilting down the table, stair descending mode. The stair descending mode starts from 30 cadence/minute and can be adjusted to 35, and the most strenuous 40 cadence/minute stair descending mode (Fig. 1).

In biometric data control group, the intensity of the rehabilitation was adjusted using heart rate or RPE. The exercise intensity was raised to the next level when the patient’s heart rate is less than target heart rate, or the RPE was less than 12 points. The target heart rate was calculated by adding 40% of HRR to resting heart rate. The reason why we set 40% of heart rate reserve is that it is low submaximal exercise intensity^[10]^ Borg’s RPE, which uses a subjective rating from 6 to 20, is a widely used tool to assess an individual’s perceived exercise intensity, and the rating of perceived scale 12 corresponds light exercise intensity.^[11]^ On the other hand, in therapist control group, the therapist comprehensively determined the patient’s response and adaptability and controlled the intensity of robotic rehabilitation. In both groups, physiological signals including heart rate were recorded from subjects when changing the intensity. The patient’s function was evaluated at baseline and at the end of therapy sessions by skilled physical therapist.

### 2.4. Functional evaluation

The primary outcome was functional ambulation category (FAC). The FAC is a common clinical gait assessment scale which distinguishes 6 levels of walking ability on the basis of the amount of physical support required.^[12]^ The secondary outcome included modified Barthel index (MBI), Berg balance scale (BBS), time up and go test, and 10 meter walk test (10MWT). MBI is a scoring system that measures the patient’s performance in 10 activities of daily life from lowest score 0, representing totally dependent bed-ridden status to 100, independent.^[13]^ BBS is 14-item scale that quantitatively measure the risk of fall and balance, calculating out of 56 possible points.^[14]^ Timed up and go test (TUG) test is a simple functional mobility test that counts time and requires a subject to stand up, walk 3 meters, turn, walk back, and sit down.^[15]^ 10MWT is short distance walking test which evaluate patients’ comfortable walking speed where they are instructed to walk 10 meters and total time is recorded.^[16]^ A licensed physiotherapist conducted all evaluation, and monitored patients for safety and side-effects.

### 2.5. Statistical analysis

All statistical analyses were performed using SPSS statistics version 18.0 (SPSS Inc, Chicago, IL). This sample size was no calculated, because this is a pilot study. The normality was examined by performing Kolmogorov–Smirnov test. To compare the subjects’ characteristics, independent *t* tests were used for normally distributed variables, and Mann–Whitney *U* test for variables which are not. To investigate functional changes before and after training in each group, Wilcoxon signed rank test were used. The Mann–Whitney test was used to compare the functional changes between two groups. Values of *P* < .05 were considered statistically significant.

## 3. Results

**Figure 2.**
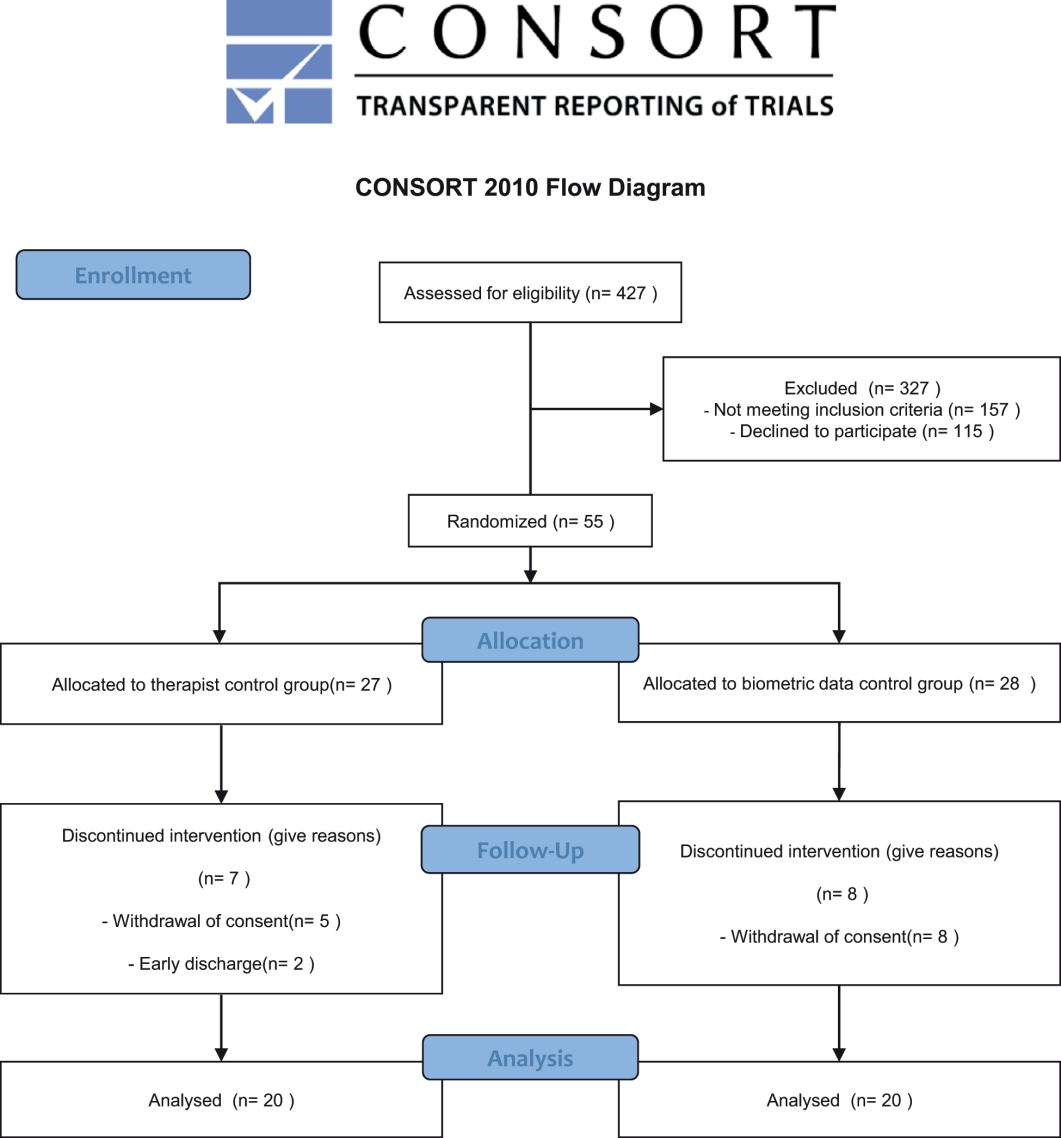
**CONSORT diagram**. 427 people were screened for eligibility, and 55 patients were enrolled. Finally, 40 patients were analyzed.

A total of 427 people were screened for eligibility from August 2018 to March 2020. Among them, 372 were excluded according to the exclusion criteria, 55 patients were enrolled. In total, 40 patients were finally analyzed: 20 in the biometric data intensity change group and 20 in the therapist control group (Fig. 2).

The characteristics of the participants are presented in Table 1. The mean age was 59.6 ± 8.95 in control group, and 64.45 ± 14.51 in case group. The mean time from stroke onset in month was 2.7 ± 3.76 in control group, and 1.6 ± 2.16 in case group. There was no significant difference in age, sex ratio, weights, heights, duration from onset between two groups (Table 1).

**Table 1:** Participant characteristics.

Variable		Therapist control group (n = 20)	Biometric data control group (n = 20)	*P* value
Age (yr)		59.60 (8.95)	64.45 (14.51)	.211
Sex (%)	Male	11 (55.0)	15 (75.0)	.32
	Female	9 (45.0)	5 (25.0)	
Weight (kg)		63.10 (10.90)	65.60 (8.92)	.432
Height (m)		1.63 (0.08)	1.66 (0.08)	.21
Time.post.stroke. (mo)		2.70 (3.76)	1.60 (2.16)	.264
Stroke.type, n (%)	Infarction	13 (65.0)	15 (75.0)	.73
	Hemorrhage.	7 (35.0)	5 (25.0)	

Values are shown as mean (%).

*P < .05. For the statistical analyses, the chi-square test and *t* tests were performed.

The functional outcome in each group was measured before the treatment and after a series of robot rehabilitation. In both groups, the primary outcome, FAC progressed significantly at the end of training, when compared to the baseline (Table 2). In therapist control group before the treatment, the median was 4.00 (interquartile range [IQR] 2.00–5.00), and it progressed to 5.00 (IQR3.00–5.00) (*P* = .031). In biometric data control group, the median of FAC before treatment was 3.00 (IQR2.00–5.00), and it also improved to 4.00(IQR3.00–5.00) (*P* = .004). Also, all the other functional measurements improved after 3 weeks. For example, in therapist control group, MBI improved from 69.00 (IQR37.75–89.25) to 95.00 (IQR60.75–98.00) (*P* = .001), and in biometric data control group, from 38.50 (IQR5.00–52.00) to 51.50 (IQR23.50–56.00) (*P* < .001). BBS score improved after robot assisted gait training. In addition, TUG and 10MWT which measure time patients ambulate significantly decreased.

**Table 2:** Functional changes at baseline and week 3.

	Therapist control group			Biometric data control group		
	Baseline	Week 3	*P* value	Baseline	Week 3	*P* value
FAC	4.00 [2.00, 5.00]	5.00 [3.00, 5.00]	.031	3.00 [2.00, 5.00]	4.00 [3.00, 5.00]	.004
MBI	69.00 [37.75, 89.25]	95.00 [60.75, 98.00]	.001	58.00 [39.50, 93.00]	88.00 [59.50, 98.00]	<.001
BBS	38.50 [5.00, 52.00]	51.50 [23.50, 56.00]	<.001	32.50 [11.75, 44.75]	46.00 [26.25, 52.50]	<.001
TUG	9.98 [8.46, 12.08]	8.03 [7.50, 8.89]	.002	15.34 [12.57, 21.83]	10.80 [9.55, 13.17]	<.001
10MWT	6.20 [5.26, 7.35]	5.18 [4.80, 5.58]	.005	9.75 [6.56, 14.62]	6.18 [4.69, 9.11]	<.001

Values are shown as median, and interquartile range are in square brackets.

For the statistical analyses, the Wilcoxon signed-rank test was performed.

10MWT = 10-meter walk test, BBS = Berg balance scale, FAC = functional ambulation category, MBI = modified Barthel index, TUG = timed up and go test.

We calculated the differences of the outcome measures of baseline and week 3 and compared them. The change of functional outcome measure did not differ significantly between the two groups (Table 3). The change of difference of primary outcome, FAC, has no significant difference between two groups (*P* = .227). In other measure such as MBI, BBS, TUG, 10MWT, the change did not show significant difference. The median of change of MBI in therapist control group was 10.00 (IQR 1.50–23.50), and 16.00 (IQR 4.00–23.00) in biometric data control group, and they have no significant difference (*P* = .447).

**Table 3:** Comparison of functional changes between the therapist control group and biometric data control group.

Variable	Therapist control group (n = 20)	Biometric data control group (n = 20)	*P* value
diff.FAC	0.00 [0.00, 1.00]	0.50 [0.00, 1.00]	.227
diff.MBI	10.00 [1.50, 23.50]	16.00 [4.00, 23.00]	.447
diff.BBS	8.00 [1.75, 13.25]	6.50 [5.00, 12.25]	.482
diff.TUG	−1.38 [−2.90, −0.89]	−5.60 [−10.05, −1.92]	.114
diff.10MWT	−0.80 [−2.17, −0.18]	−2.24 [−4.21, −1.37]	.061

Values are shown as median, and interquartile range are in square brackets.

For the statistical analysis, the Mann–Whitney *U* test was performed.

10MWT = 10-meter walk test, BBS = Berg balance scale, Diff, the difference the outcome measure of week 3 and baseline (week 3-basline), FAC = functional ambulation category, MBI = modified Barthel index, TUG = timed up and go test.

We recorded counts of intensity adjustments in each training sessions, in both groups, the number of intensity setting did not show significant difference. The median of number of changes in therapist control group was 4.00 (IQR 2.00–7.00 n = 20), and 4.00 (IQR 4.00–5.00, n = 19) in biometric data control group (*P* = .887).

When comparing heart rate between therapist control and biometric data control group, the median of heart rate in therapist control group was significantly higher, 106.00 (IQR 73.00–139.00) than in biometric data control group, 96.00 (IQR 64.00–134.00) (*P* < .001) (Table 4).

**Table 4:** Comparison of median number of intensity changes between therapist control group and biometric data control group.

Variable	Therapist control	Biometric data control	*P* value
n	20	19	
Number of changes	4.00 [2.00, 7.00]	4.00 [3.00, 5.00]	.887

Values are shown as median, and interquartile ranges are in square brackets.

19 patients were analyzed in biometric data control group due to missing data.

*P < .05. For the statistical analysis, the Mann–Whitney *U* test was performed.

## 4. Discussion

This study shows that using biometric data on intensity control at robot-assisted gait training was effective to functional recovery of patient. And compared to the changing intensity according to subjective judgment of therapist, which is conventional therapy at this point, it did not show significant difference in patients’ gait function and balance. To the extent of our knowledge, it is the first preliminary study to investigate the possibilities to contrive artificial intelligence protocol which can adjust intensity of robot-assisted gait training using patients’ biometric data.

The clinical effectiveness of Morning Walk® as a gait training end effector–type robot was proved in previous study. However, in the previous study, the intensity of the robot assisted gait training was controlled only by the therapist.^[6]^ In contrast, in this research, intensity was adjusted using biometric data and the therapist’s judgement. And all measured functional outcomes including FAC, BBS, TUG, 10MWT improved after the rehabilitation program. The result agrees with the previous study and suggests not only that robot-assisted gait training is effective for gait recovery of stroke patient, but also gait training protocol based on patient’s heart rate or RPE is beneficial.

There have been few attempts to control and monitor gait rehabilitation intensity in stroke. Macko et al^[17]^ elucidated the positive effect of controlling intensity based on heart rate in locomotor training on walking recovery poststroke. Gait training on targeted intensity can elicit neural excitability that underlies improved walking ability.^[18,19]^ In the studies, the gait training was therapist assist gait training. There has been no previous studies intensity control in robot assisted gait training, and this study showed that using the patient’s heart rate or RPE, the treatment effect was similar to that of the judgment of the therapist. The change before and after the robot assisted gait training has no significant difference between two groups. This can suggest that intensity control based on biometric data has comparable effects as the conventional method, therapist intensity control.

Recent studies found that effective neurorehabilitation need to be highly repetitive, and task-oriented.^[20,21]^ In therapy control group, the therapist should continuously interact with the patient changing the intensity, and this may promote the concentration of the patient. Without continuous feedback, patients in biometric intensity control group might feel bored and it may interfere with the task-oriented gait training, which result in ineffectiveness. Winstein reported that stroke survivors provided with visual information when had better balancing ability than those who received conventional physical therapy.^[22]^ By providing continuous visualized feedback, the limitation of robotic rehabilitation can be compensated. The Morning Walk ® provides the graphical information on the monitor as visual biofeedback to the patient for forming good center of pressure pattern.^[6]^

The number of intensities change in both groups did not show significant difference. This suggests that intensity adjusting based on patients’ biometric data has functionally no difference and therefore it can be applied to in clinical setting instead of therapist’s intensity control. When comparing the median heart rate of two group when changing the intensity, the median of heart rate was significantly higher in therapist control group than in biometric data control group. It may suggest that the intensity is controlled more sensitively in biometric data control group than in therapist control group. In therapist control group, the therapist comprehensively interprets the response of patient and control the intensity, and it is difficult to respond to small change in heart rate or RPE. However, in biometric data group, the heart rate and RPE is continuously monitored, so it can change the intensity more sensitively and immediately.

This study has several limitations. First, the sample size is small, so the results can be biased. However, this is a pilot study, and further research is needed in larger sample size.

Also, patients and the researchers knew which group there were allocated, so it might also affect the results. This study’s internal validity can be disturbed. In the future, double-blinded study is warranted.

Finally, the subject enrolled in the study consist of heterogeneous acute, and subacute stage stroke patients. However, only 3 patients who experienced stroke over 6 months prior to the study were included, 1 in biometric data control group, and 2 in therapist control group. Only small number of subacute stage so it would not significantly affect the functional evaluation outcome. Individual study on each stage would be helpful, we hope that, in the future, more specific study will be conducted.

## 5. Conclusion

When the robot intensity was adjusted using the patient’s heart rate or RPE, the treatment effect has no significant difference to when adjusting the intensity according to the know-how of the therapist. These results show the possibility that artificial intensity adjustment protocol can be contrived at robotic rehabilitation. Further study is needed to develop protocol of robot-assisted gait training in patients with stroke.

## Author contributions

**Conceptualization:** Min Ho Chun, Jun Won Kim, Ji Yeon Lee.

**Data curation:** Kim Jiae, Jun Won Kim, Ji Yeon Lee.

**Formal analysis:** Kim Jiae, Jun Won Kim.

**Funding acquisition:** Min Ho Chun.

**Investigation:** Min Ho Chun, Junekyung Lee, Jun Won Kim, Ji Yeon Lee.

**Project administration:** Kim Jiae, Ji Yeon Lee.

**Resources:** Junekyung Lee.

**Supervision:** Junekyung Lee.

**Validation:** Min Ho Chun.

**Visualization:** Kim Jiae, Min Ho Chun.

**Writing – original draft:** Kim Jiae.

**Writing – review & editing:** Kim Jiae, Min Ho Chun.

## References

[1] Chang WH Kim YH. Robot-assisted therapy in stroke rehabilitation. J Stroke. 2013;15:174–81. 24396811 10.5853/jos.2013.15.3.174 PMC3859002

[2] Koenig A Novak D Omlin X. Real-time closed-loop control of cognitive load in neurological patients during robot-assisted gait training. IEEE Trans Neural Syst Rehabil Eng. 2011;19:453–64. 21827971 10.1109/TNSRE.2011.2160460

[3] Duschau-Wicke A Caprez A Riener R. Patient-cooperative control increases active participation of individuals with SCI during robot-aided gait training. J Neuroeng Rehabil. 2010;7:43. 20828422 10.1186/1743-0003-7-43 PMC2949707

[4] Schwartz I Sajin A Fisher I. The effectiveness of locomotor therapy using robotic-assisted gait training in subacute stroke patients: a randomized controlled trial. PMR. 2009;1:516–23. 10.1016/j.pmrj.2009.03.009 19627940

[5] Mayr A Kofler M Quirbach E. Prospective, blinded, randomized crossover study of gait rehabilitation in stroke patients using the Lokomat gait orthosis. Neurorehabil Neural Repair. 2007;21:307–14. 17476001 10.1177/1545968307300697

[6] Kim J Kim DY Chun MH. Effects of robot-(Morning Walk®) assisted gait training for patients after stroke: a randomized controlled trial. Clin Rehabil. 2019;33:516–23. 30326747 10.1177/0269215518806563

[7] Morone G Paolucci S Cherubini A. Robot-assisted gait training for stroke patients: current state of the art and perspectives of robotics. Neuropsychiatr Dis Treat. 2017;13:1303–11. 28553117 10.2147/NDT.S114102 PMC5440028

[8] Foley NC Teasell RW Bhogal SK. The efficacy of stroke rehabilitation: a qualitative review. Top Stroke Rehabil. 2003;10:1–18. 10.1310/aqe1-pcw1-fw9k-m01g 13680515

[9] Colberg SR Swain DP Vinik AI. Use of heart rate reserve and rating of perceived exertion to prescribe exercise intensity in diabetic autonomic neuropathy. Diabetes Care. 2003;26:986–90. 12663561 10.2337/diacare.26.4.986

[10] Panton LB Graves JE Pollock ML. Relative heart rate, heart rate reserve, and VO_2_ during submaximal exercise in the elderly. J Gerontol A Biol Sci Med Sci. 1996;51:M165–71. 8680999 10.1093/gerona/51a.4.m165

[11] Scherr J Wolfarth B Christle JW. Associations between Borg’s rating of perceived exertion and physiological measures of exercise intensity. Eur J Appl Physiol. 2013;113:147–55. 22615009 10.1007/s00421-012-2421-x

[R12] [12]Mehrholz J Wagner K Rutte K. Predictive validity and responsiveness of the functional ambulation category in hemiparetic patients after stroke. Arch Phys Med Rehabil. 2007;88:1314–9. 17908575 10.1016/j.apmr.2007.06.764

[13] Sulter G Steen C De Keyser J. Use of the Barthel index and modified Rankin scale in acute stroke trials. Stroke. 1999;30:1538–41. 10436097 10.1161/01.str.30.8.1538

[14] Blum L Korner-Bitensky N. Usefulness of the Berg Balance Scale in stroke rehabilitation: a systematic review. Phys Ther. 2008;88:559–66. 18292215 10.2522/ptj.20070205

[15] Ng SS Hui-Chan CW. The timed up & go test: its reliability and association with lower-limb impairments and locomotor capacities in people with chronic stroke. Arch Phys Med Rehabil. 2005;86:1641–7. 16084820 10.1016/j.apmr.2005.01.011

[16] Dalgas U Severinsen K Overgaard K. Relations between 6 minute walking distance and 10 meter walking speed in patients with multiple sclerosis and stroke. Arch Phys Med Rehabil. 2012;93:1167–72. 22421626 10.1016/j.apmr.2012.02.026

[17] Macko RF Ivey FM Forrester LW. Treadmill exercise rehabilitation improves ambulatory function and cardiovascular fitness in patients with chronic stroke: a randomized, controlled trial. Stroke. 2005;36:2206–11. 16151035 10.1161/01.STR.0000181076.91805.89

[18] Luft AR Macko RF Forrester LW. Treadmill exercise activates subcortical neural networks and improves walking after stroke: a randomized controlled trial. Stroke. 2008;39:3341–50. 18757284 10.1161/STROKEAHA.108.527531 PMC2929142

[19] Yen CL Wang RY Liao KK. Gait training induced change in corticomotor excitability in patients with chronic stroke. Neurorehabil Neural Repair. 2008;22:22–30. 17507641 10.1177/1545968307301875

[20] Bütefisch C Hummelsheim H Denzler P. Repetitive training of isolated movements improves the outcome of motor rehabilitation of the centrally paretic hand. J Neurol Sci. 1995;130:59–68. 7650532 10.1016/0022-510x(95)00003-k

[21] Bayona NA Bitensky J Salter K. The role of task-specific training in rehabilitation therapies. Top Stroke Rehabil. 2005;12:58–65. 10.1310/BQM5-6YGB-MVJ5-WVCR 16110428

[22] Winstein CJ Gardner ER McNeal DR. Standing balance training: effect on balance and locomotion in hemiparetic adults. Arch Phys Med Rehabil. 1989;70:755–62. 2802955

